# Cystorrhœa vs. Matrimony

**Published:** 1861-11

**Authors:** 


*Cystorrhœa vs. Matrimony.*—A correspondent of the Nashville *Journal of Medicine and Surgery*, in the May number of that Journal says, that for two years he was troubled with cystitis. Several "eminent physicians" were consulted, and a variety of treatment brought to bear upon the case, among which were injections into the bladder of a solution of nitrate of silver, twenty grains to the ounce.

He was advised by his physician not to marry; but, after being treated ineffectually for two years, he disregarded the advice in this particular. He says: "In less than three weeks after I married, the disease was entirely well, and I have had but very few slight symptoms of the affection since, (now, about a year.) It seemed to reduce all excitement and produce an equilibrium in the system that acted like a charm."

We think the *non-professional* treatment would be less disagreeable than injections into the bladder of a solution of nitrate of silver, twenty grains to the ounce! But should a married man unfortunately be afflicted with cystitis, what is the remedy? Must he marry again?—*St. Louis Medical and Surgical Journal*.

